# Understanding non-stationarity of hydroclimatic extremes and resilience in Peninsular catchments, India

**DOI:** 10.1038/s41598-023-38771-w

**Published:** 2023-08-02

**Authors:** Nikhil Kumar, Piyush Patel, Shivam Singh, Manish Kumar Goyal

**Affiliations:** Department of Civil Engineering, Indian Institute of Technology, Indore, 453552 India

**Keywords:** Atmospheric science, Climate change, Hydrology, Climate sciences, Hydrology

## Abstract

Climate change significantly impacts the global hydrological cycle, leading to pronounced shifts in hydroclimatic extremes such as increased duration, occurrence, and intensity. Despite these significant changes, our understanding of hydroclimatic risks and hydrological resilience remains limited, particularly at the catchment scale in peninsular India. This study aims to address this gap by examining hydroclimatic extremes and resilience in 54 peninsular catchments from 1988 to 2011. We initially assess extreme precipitation and discharge indices and estimate design return levels using non-stationary Generalized Extreme Value (GEV) models that use global climate modes (ENSO, IOD, and AMO) as covariates. Further, hydrological resilience is evaluated using a convex model that inputs simulated discharge from the best hydrological model among SVM, RVM, random forest, and a conceptual model (*abcd*). Our analysis shows that the spatial patterns of mean extreme precipitation indices (R1 and R5) mostly resemble with extreme discharge indices (Q1 and Q5). Additionally, all extreme indices, including R1, Q1, R5, and Q5, demonstrate non-stationary behavior, indicating the substantial influence of global climate modes on extreme precipitation and flooding across the catchments. Our results indicate that the random forest model outperforms the others. Furthermore, we find that 68.52% of the catchments exhibit low to moderate hydrological resilience. Our findings emphasize the importance of understanding hydroclimatic risks and catchment resilience for accurate climate change impact predictions and effective adaptation strategies.

## Introduction

Projected impacts of hydroclimatic extremes pose implications in terms of risk and resilience at a catchment scale. By evaluating extremes in precipitation and discharge, an enhanced understanding of climate change's potential effects can be achieved, since precipitation is the main input hydrological process, while discharge represents an aggregated response of different hydroclimatic variables at a catchment scale^1,2^. In the face of severe disturbances such as floods and droughts due to climate change^3,4^, it's critical to comprehend the resilience capacity of catchments, specifically, the magnitude of disturbances they can resist and recover from. Therefore, catchment scale studies are crucial for assessing the impacts of climate change on catchment resilience and hydroclimatic extremes. These studies can play a key role in evaluating the risk and vulnerability of water resource systems.

Many studies have applied the Extreme Value Theory (EVT) to evaluate the risks associated with extreme rainfall or flood events^5,6^. The EVT involves a two-step process. Initially, extreme events, such as floods, are isolated using methods like block maxima or the Peaks Over Threshold method. Subsequently, a theoretical probability distribution, such as the Generalized Extreme Value or the Generalized Pareto distribution, is fitted to this separated series of extreme events. From this fitted distribution, the magnitude of the required design discharge is determined in order to assess the associated risk. Traditionally, many studies have adopted a stationary approach, where the parameters of the distribution fitted using EVT are assumed to remain constant over time^7^. However, with the ever-evolving climate and its dynamic effects on the hydroclimatic system, this stationary approach may no longer suffice for risk assessment^8^. Non-stationarities introduced by climatic and anthropogenic changes could lead to substantial errors in estimating return levels of extreme^9^. These inaccuracies can result in significant misestimations, either underestimating or overestimating the likelihood of extreme events, thus misrepresenting the associated risk. For instance, the 2013 Boulder, Colorado flood exceeded stationary model predictions, underestimating flood risk^10^. This indicates the need for non-stationary approaches, balancing risk predictions while acknowledging the inherent uncertainties^8,11^. To fulfill this gap, a non-stationary approach is incorporated in modelling extreme events using EVT. Recently, several studies are conducted using a non-stationary approach to understand behaviour of extremes^12,13^.

It's widely recognized that global climate modes, which include large-scale atmospheric oscillations such as ENSO, IOD, and AMO, have a significant relationship with hydroclimatic extremes^12,14,15^. Understanding these linkages can notably enhance strategies to manage these extremes. Within the Indian region, a substantial amount of research has been carried out to explore these connections. Some studies have indicated an increasing influence of ENSO on the north-eastern monsoon^16^ and winter precipitation^17^. Furthermore, cool and warm ENSO phases have been associated with excess and deficit precipitation across India, respectively^18^. For example, El Niño events often lead to severe droughts in Indian Peninsular region, such as in 2002 and 2009^19,20^. Conversely, La Niña events result in above-average rainfall, as experienced during the major floods in 2007 and 2010^21,22^. One of the other global climate modes i.e., IOD (Indian Ocean Dipole) is also found to influence the monsoon precipitation pattern over India^23^, where positive IOD is linked with excess precipitation in northern India^24^. In 2019, a strong positive IOD event contributed to heavy monsoon rainfall and widespread flooding, particularly in Maharashtra, Karnataka, and Kerala^25^. Conversely, a negative IOD event in 2010 was associated with deficient monsoon rains and drought conditions in many parts of the peninsular region^26^. Furthermore, another global climate mode i.e., AMO also impact summer monsoon precipitation^27^ and can explain the decadal variability of this season^28^. Based upon the above discussion, it can be concluded that ENSO, IOD and AMO are the major global climate modes, influencing precipitation patterns across peninsular India. Thus, accurate evaluation of myriad dimensions of these linkages will be crucial in order to minimize risk due to extreme precipitation and floods. Therefore, in the present study, global climate modes are taken as co-variates to model non-stationary GEV models of extreme precipitation and discharge indices, for reliable estimation of return levels.

In India, the severity of floods and droughts has been increasing in recent decades and is further projected to rise in the future^18,29,30^. Floods account for over half of the losses incurred in climate-related disasters in India^31^. A recent study indicates that extreme precipitation events (daily rainfall ≥ 150 mm) have increased three times during 1950–2015 over central India^18^. While there are many studies focused on extreme precipitation events, literature on floods is scarce and information about how these extreme precipitation events translate to floods at a catchment scale is limited over India. Moreover, such disturbances lead to hydro-ecological impacts on catchments and therefore can alter the functioning of catchments and affect water resource systems. Though microclimate and physiological factors significantly influence local hydroclimatic responses^32,33^, this study aims to identify broader catchment-scale trends. Such a scale provides pivotal insights for macro-level strategies in water management and climate change adaptation^34,35^. However, local microclimatic and physiological variations may not be fully captured in catchment-scale studies, a potential limitation that future research could address to enrich our understanding of hydroclimatic dynamics.

Therefore, this study sets out to comprehensively understand hydroclimatic extremes and resilience at a catchment scale. First, a spatiotemporal assessment of extreme precipitation and discharge indices is carried out across 54 peninsular catchments for the period 1988–2011. Second, investigation of risk due to extreme precipitation/discharge indices with the implementation of non-stationary GEV models using covariates as ENSO, IOD and AMO. Third, machine learning models (SVM, RVM and random forest) and a conceptual water balance model are calibrated at a monthly scale for discharge estimation across 54 peninsular catchments during 1988–2011. Furthermore, simulated discharge from the best-performing model is used to quantify the catchment resilience using a convex model.

## Results

### Hydroclimatic extreme indices

#### Mean and trend analysis

**Figure 1:**
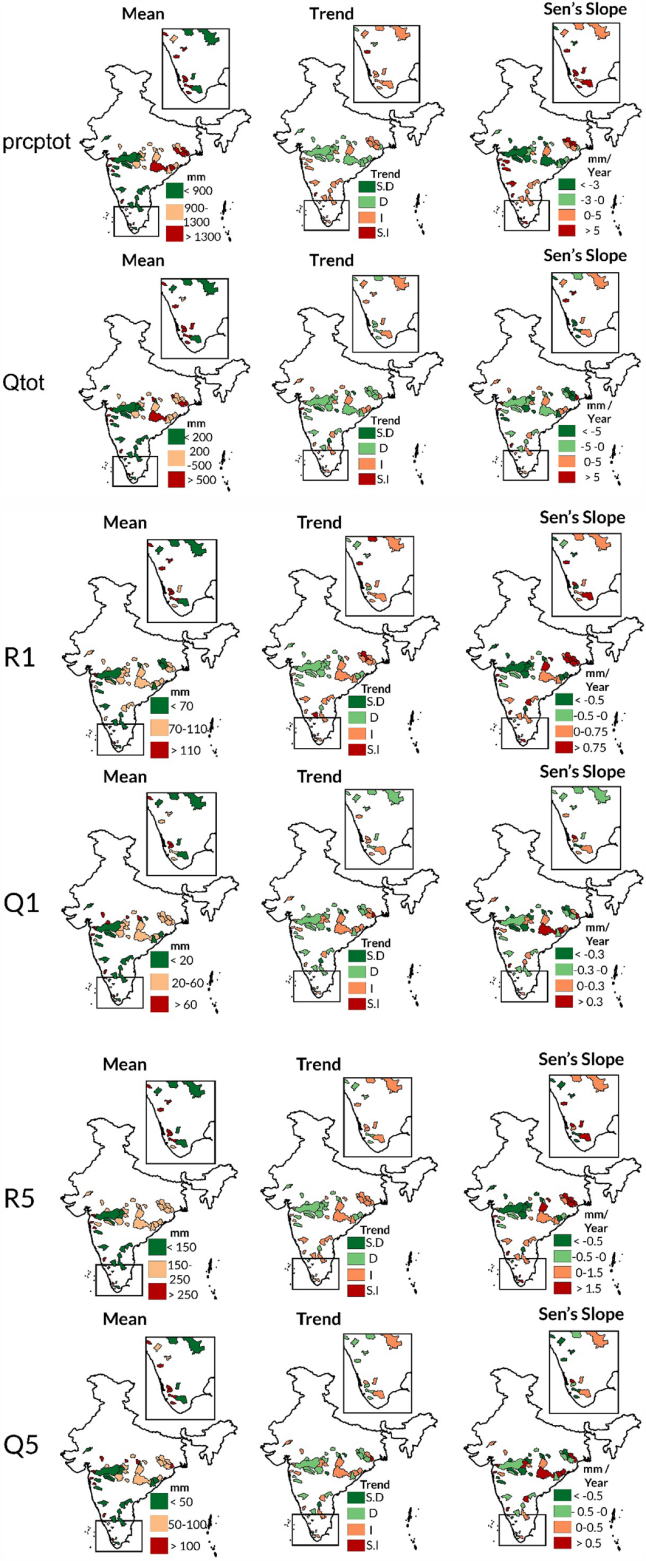
Spatial pattern of mean, trend and Sen’s slope of hydroclimatic extreme indices (Prcptot, Qtot, R1, Q1, R5 and Q5) of 54 catchments in peninsular India during 1988–2011. The maps were created using ArcGIS 10.7 software (https://www.esri.com/en-us/arcgis/products/arcgis-desktop/resources).

Before the non-stationary investigation of hydroclimatic extremes, a preliminary analysis of extremes is carried out using mean and trend analysis (Mann Kendall test at 95% confidence interval or 5% level of significance and Sen’s Slope estimator) for peninsular catchments during 1988–2011^36,37^. The results show that the mean prcptot (annual precipitation) is low (< 900 mm), moderate (900–1300 mm) and high (> 1300 mm) in 33.33% of catchments each. Moderate to high mean prcptot primarily characterizes central-eastern, southernmost Western Ghats, and parts of the western region. On the other hand, central and southeastern regions predominantly exhibit low prcptot values (Fig. 1). Trend analysis reveals a positive trend in 53.70% of catchments, with a magnitude of 0–5/> 5 mm/year in 24.07/29.63% catchments, predominantly lying in upper central-eastern, western ghats and southern region. Conversely, 46.30% of catchments show a negative trend, with 18.52/27.78% having a trend magnitude of < − 3/− 3–0 mm/year, mainly in central and lower central-eastern regions. Notably, the spatial distribution of mean qtot closely resembles that of mean prcptot. Mean qtot also shows low (< 200 mm), moderate (200–500 mm), and high (> 500 mm) in 33.33% catchments each, identical as mean prcptot. The central, central-eastern, southernmost western ghats and parts of western region mostly exhibit a moderate-high mean qtot, whereas parts of southern-eastern region indicate a lower mean qtot. Trend analysis shows a positive trend in 40.74% of catchments, with a rate of 0–5/ > 5 mm/year in 27.78/12.96% catchments, primarily in small catchments. On the other hand, 59.26% of catchments show a negative trend, with 33.33/25.93% catchments having a trend magnitude of < − 5/− 5–0 mm/year, dispersed across the study area. Central and central-eastern regions showcase a few catchments with a negative trend in both prcptot and qtot. Overall, the spatial patterns identified in the trend analysis of prcptot and qtot slightly resemble each other.

Around 70.37% of the catchments record moderate to high mean R1 (53.70% fall within the range of 70–110 mm and 16.67% exceed 110 mm), predominantly located in central, centre-eastern, and southern Western Ghats, and some in the western region. The remaining catchments (29.63%) exhibit low mean R1 (> 70 mm), mostly situated in the south-eastern and parts of the central region. The R1 trend is positive in 51.85% of catchments, with 20.37% showing a trend magnitude of 0–0.75 mm/year and 31.48% exceeding 0.75 mm/year, mostly in the central-eastern and southernmost regions. On the other hand, R1 shows a negative trend in 48.15% of catchments, with magnitudes of < − 0.5 mm/year for 31.48% and between − 0.5 to 0 mm/year for 16.67% catchments, largely in the central region. The spatial pattern of mean Q1 closely aligns with that of mean R1. Around 62.96% of catchments show moderate to high magnitude Q1 (42.59% between 20–60 mm and 20.37% exceeding 60 mm), primarily in central, central-eastern regions, parts of southern Western Ghats, and a few catchments in the western region. The remaining 37.04% of catchments have a low mean Q1 (< 20 mm), typically moderate-large sized catchments in central and south-eastern regions. The Q1 trend is positive in 40.74% of catchments (25.93% showing 0–0.3 mm/year and 14.81% exceeding 0.3 mm/year), primarily in central-eastern and a few southernmost catchments. The remaining 59.26% of catchments exhibit a negative trend, with magnitudes of < − 0.3 mm/year for 29.63% and between − 0.3 to 0 mm/year for 29.63%, mostly in the central region. Overall, the spatial distribution of R1 and Q1 trends closely resemble each other.

In 74.07% of the catchments, the mean R5 ranges from moderate to high (53.70% between 150–250 mm and 20.37% exceeding 250 mm), primarily located in the centre-eastern, southernmost Western Ghats, and certain parts of the western region. The remaining catchments (25.93%) show low mean R5 (< 150 mm), mostly in the central and parts of the south-eastern region. Trend analysis indicates a positive trend in 53.70% of catchments, with 25.93% showing a trend magnitude of 0–1.5 mm/year and 27.78% exceeding 1.5 mm/year, predominantly in the southernmost parts and centre-eastern region. A negative trend is observed in 46.30% of catchments, with magnitudes of < − 0.5 mm/year for 31.48% and between − 0.5 to 0 mm/year for 14.81% of catchments, primarily in the central region. The mean Q5 exhibits a spatial pattern almost identical to mean R5, with 35.19% of catchments showing a low value (< 50 mm), 37.04% moderate (50–100 mm), and 27.78% high (> 100 mm). Additionally, 40.74% of catchments show a positive trend (16.67% recording 0–0.5 mm/year and 24.07% exceeding 0.5 mm/year), mostly in small to moderate-sized catchments. The remaining 59.26% show a negative trend, with magnitudes of < − 0.5 mm/year for 37.04% and between − 0.5 to 0 mm/year for 22.22% of catchments, predominantly in the southernmost, central, and parts of upper centre-eastern regions. In general, R5 and Q5 trends exhibit high resemblance except in parts of central-eastern region.

#### Non-stationary assessment

In this section, a comprehensive evaluation of hydroclimatic extremes is carried out using return levels at different time intervals (10-year, 50-year and 100-year), obtained from best model from 27 models (stationary-M0 and non-stationary (NS): M1-M26) for the period 1988–2011 (Table S2). It is noteworthy that the non-stationary models performed as the best model for 54 catchments for each hydroclimatic extreme index (R1, Q1, R5 and Q5). As non-stationary models offer advantages over stationary models in assessing extreme event risks by capturing temporal variations and trends, providing more accurate projections of future risks^38^. Our findings show that stationary models can underestimate or overestimate the risk associated with extremes based on return levels, as depicted in Fig. S1. Further, the results also show that spatial distribution of 10-year, 50-year and 100-year return levels for each hydroclimate extreme index resemble each other across the study area. Henceforth, the 10-year return levels are considered for spatial assessment of vulnerability towards a particular extreme indice.

**Figure 2:**
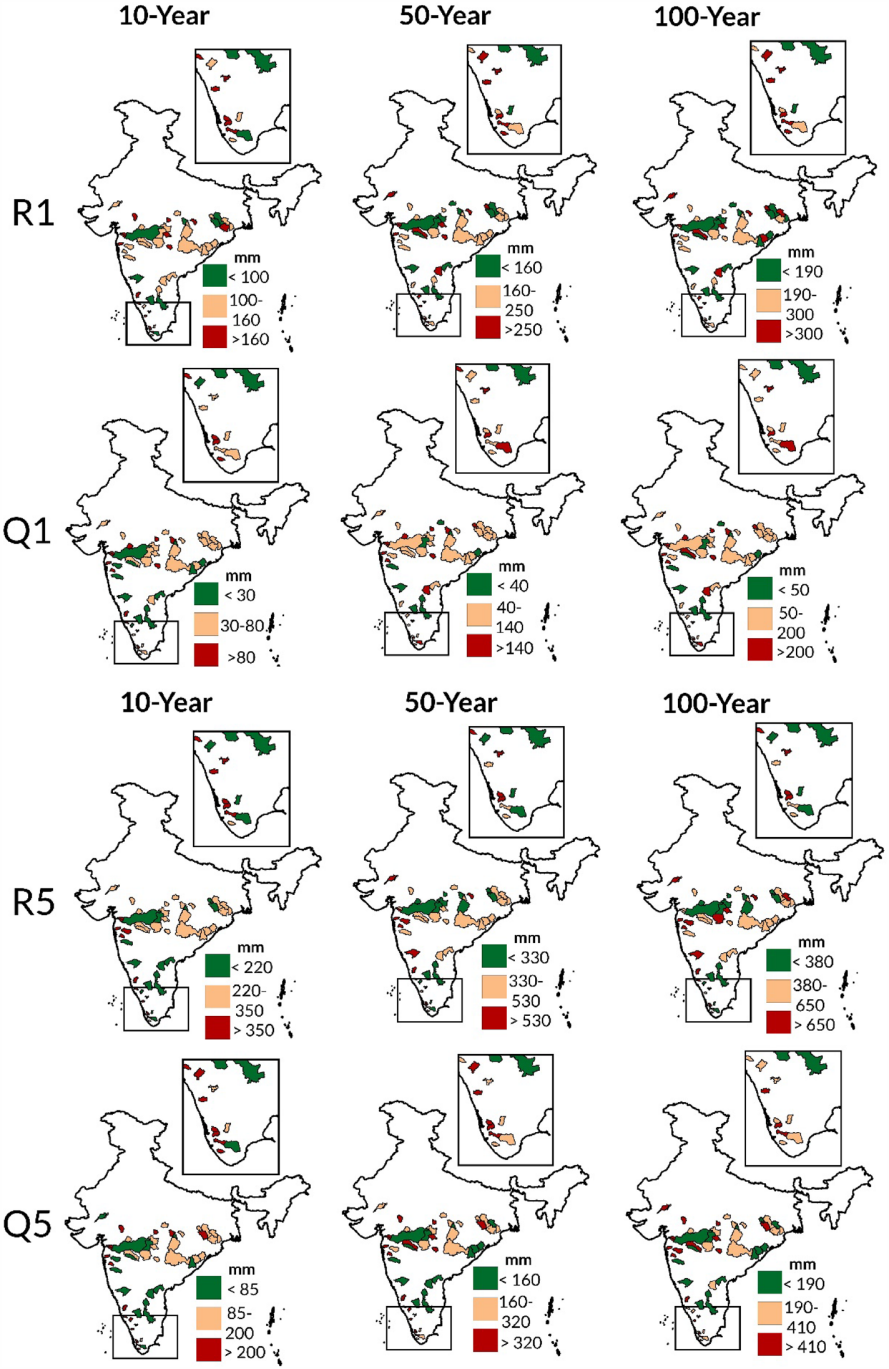
Spatial pattern of return levels (10-year, 50-year and 100-year) of hydroclimatic extremes (R1, Q1, R5 and Q5) obtained using best model from 27 GEV models. The maps were created using ArcGIS 10.7 software (https://www.esri.com/en-us/arcgis/products/arcgis-desktop/resources).

For R1, the best models are M11 (applicable to 18.52% of catchments), M14 (9.26%), and M19 (9.26%), while other NS models best represent the remaining catchments (62.96%). ^10yr^R1 represents 10-year return level of R1 during 1988–2011 obtained using the best model and similar representation is utilized for other extreme indices as well. ^10yr^R1 encompasses low (< 100 mm), moderate (100–160 mm), and high (> 160 mm) in 14.81%, 53.70% and 31.48% catchments, respectively. It indicates a similar spatial pattern as mean R1 (Fig. 2), showing a moderate-high magnitude in central, central-eastern and southern western ghats region. Additionally, ^50yr^R1and ^100yr^R1, each show high magnitude (> 250 and > 300 mm) in 29.63% catchments. As for Q1, the best models are M11 (25.93% of catchments), M7 (22.22%), and other NS models (51.85%). The spatial pattern of ^10yr^Q1 show high resemblance with ^10yr^R1, with 25.93%, 48.15% and 25.93% of catchments exhibiting low (< 30 mm), moderate (30–80 mm), and high (> 80 mm) magnitude, respectively. Similarly, ^50yr^Q1/^100yr^Q1 attain high magnitude (> 140 and > 200 mm) in 27.78% of the catchments each. Consequently, it can be inferred that the spatial patterns of mean R1, ^10yr^R1, mean Q1 and ^10yr^Q1 show resemblance with each other.

In the case of R5, the best -performing models are M11 (applicable to 12.96% of catchments) and M14 (11.11%), while other NS models are suited for the remaining catchments (75.93%). Notably, M11 and M14 also emerge as the two best fitting models for R1, based on their performance across the study area. The results indicate that ^10yr^R5 falls into low (< 220 mm), moderate (220–350 mm), and high (> 350 mm) categories in 29.63%, 48.15% and 22.22% of catchments, respectively. The spatial distribution of ^10yr^R5 is similar to that of mean R5, with the moderate-high ^10yr^R5, primarily observed in central, central-eastern, southernmost western ghats and parts of the western region, while low ^10yr^R5 is mostly found in parts of the south-eastern region. Moreover, ^50yr^R5 and ^100yr^R5 show high magnitude (> 530 and > 650 mm) in 22.22 and 25.93% of catchments respectively. For Q5, the best models identified are M7 (27.78% of catchments) and M12 (16.67%), while other NS models are best fit for the remaining catchments (55.56%).^10yr^Q5 show low (< 85 mm), moderate (85–200 mm), and high (> 200 mm) magnitude are 29.63%, 40.74% and 29.63% of catchments, respectively. The spatial distribution of ^10yr^Q5 shares a high resemblance with ^10yr^R5. It is also evident that the patterns exhibited by mean R5, ^10yr^R5, mean Q5 and ^10yr^Q5 illustrate resemblance among each other.

### Model performance

**Figure 3:**
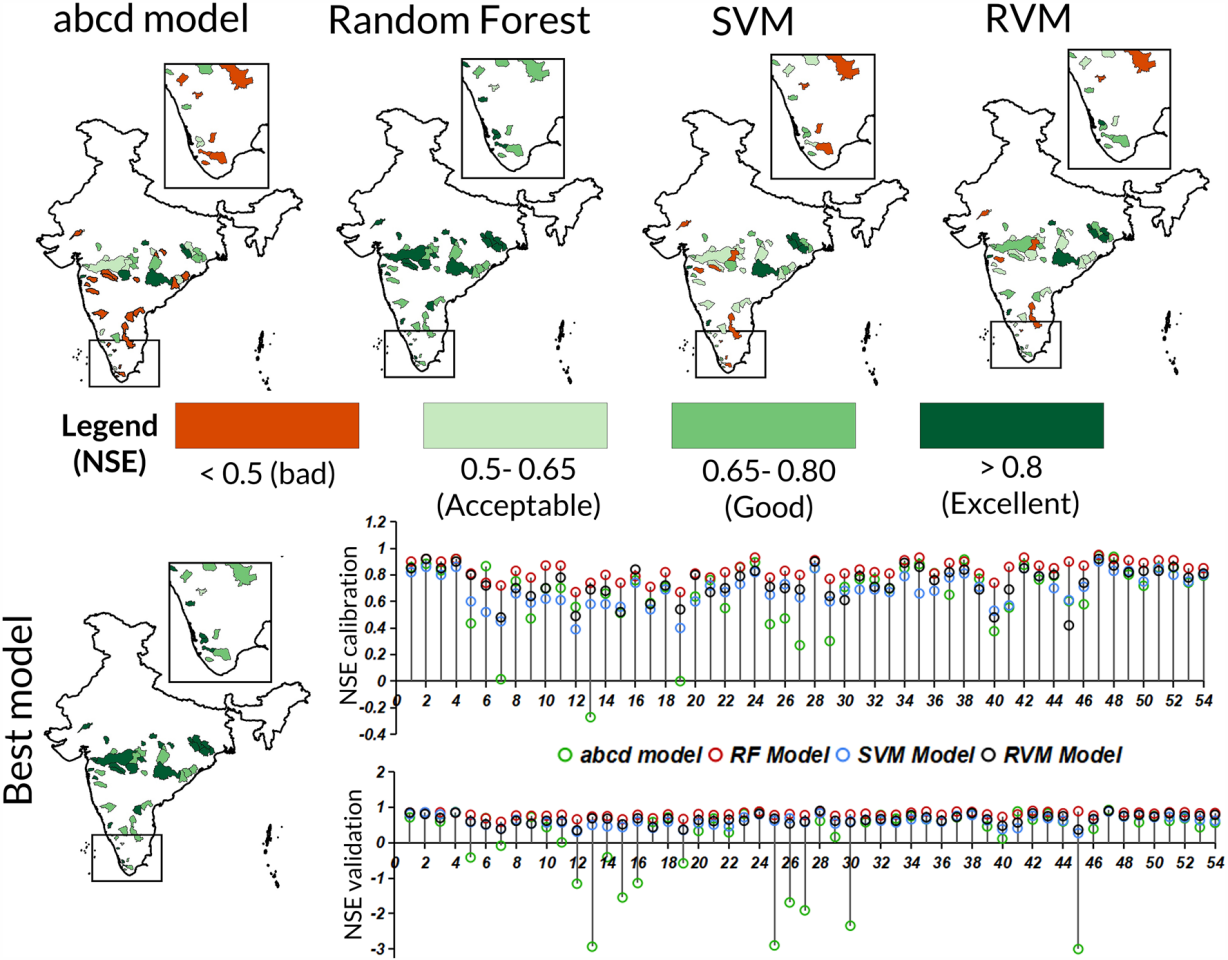
Spatial pattern of overall model performance and model performance during calibration and validation (bar chart) for abcd model, random forest, SVM and RVM during 1988–2011. The maps were created using ArcGIS 10.7 software (https://www.esri.com/en-us/arcgis/products/arcgis-desktop/resources).

The model performance is characterized in excellent (> 0.80), good (0.65–0.80), acceptable (0.5–0.65) and bad (< 0.50) categories based upon Nash Sutcliffe Efficiency (NSE). The NSE statistics has been used extensively for defining model performance^39^ and therefore enables us to compare our results with earlier studies. For *abcd* model of 54 catchments, the calibration is carried out in previous study^40^ and results are discussed here briefly. In *abcd* model, 81.5% of the catchments show more than bad performance limit (NSE > 0.50) in calibration period, however only 60% of catchments performed above this range (Fig. 3). For random forest model, all catchments performed above bad range in calibration as well in validation period. In case of SVM, 94.4% catchments show above bad performance in calibration period, whereas the number of catchments performing above bad range plummets to 81.48% in validation period. Similarly, RVM shows similar behaviour with 92.5% catchments in validation period and 88.88% catchments in calibration period performing above bad range. Furthermore, the overall performance is considered as minimum of NSE statistics in calibration and validation period and is categorized as excellent (> 0.80), good (0.65–0.80), acceptable (0.5–0.65) and bad (< 0.50) as shown in Fig. 3. Overall performance of *abcd* model indicates 57% catchments have acceptable (12), good (13) and excellent scores (6). Overall performance of SVM/RVM model are similar and indicates 81.48/88.88% catchments have acceptable (21/18), good (16/21) and excellent scores (7/9).

It is noteworthy that random forest (RF) outperforms *abcd* model by a large margin, and SVM/RVM by relatively less margin, by performing 100% above 0.50 threshold with acceptable (1), good (22) and excellent (31). This represents that machine learning models have more potential than conceptual water balance model (i.e., *abcd*) to simulate discharge at monthly scale. To conclude, random forest performance was best among the chosen models. Moreover, we selected the best-performing models (those that demonstrated the best pair of NSE in calibration and validation) for each catchment. A performance summary reveals that the best models encompassed 31 catchments; 27 with Random Forest (RF), 2 with Support Vector Machines (SVM), and 2 with Relevance Vector Machines (RVM); that delivered excellent results. Additionally, 22 catchments (all RF) performed well, and 1 catchment (RF) fell into the acceptable category.

### Catchment resilience

Resilience in a system denotes its adaptive capability to perturbations, reflecting the scale of disturbance a system can resist and recover from^40,41^. The present study uses a systematic approach based on the principle of critical slowing down and convex model for temporal assessment of catchment resilience. Here we use annual discharge as state variable for evaluation of catchment resilience, as dams and other anthropogenic activities can induce significant alterations in hydrological flow of rivers at shorter time scale (daily/monthly/seasonal)^42^. A resilient catchment possesses the capacity to return to equilibrium after disturbances, exemplified by how the magnitude of annual discharge adjusts following extreme weather events such as floods or droughts.

**Figure 4:**
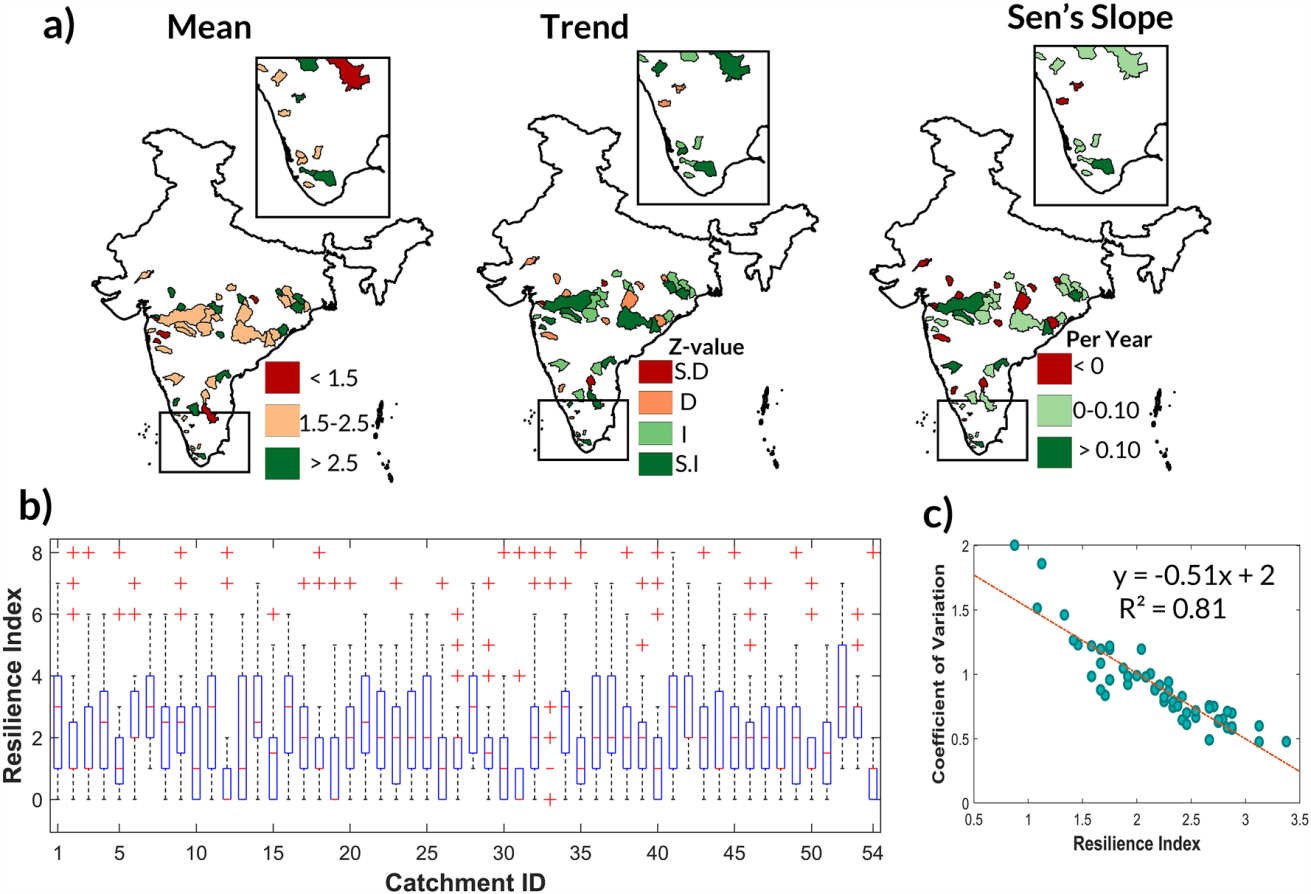
(**a**) Spatial pattern of mean resilience index, trend and Sen’s slope (**b**) Box-plot of resilience index (**c**) Relation between mean and coefficient of variation resilience index of 54 catchments in peninsular India during 1988–2011 (Z-value suggest S.D—significantly decreasing, D—decreasfing, I—increasing and S.I—significantly increasing at 95% confidence interval). The maps were created using ArcGIS 10.7 software (https://www.esri.com/en-us/arcgis/products/arcgis-desktop/resources).

Utilizing monthly discharge simulations from best models, an annual discharge time series is constructed for 1988–2011 to calculate resilience index. Catchments are classified as low (< 1.5), moderate (1.5 to 2.5) and highly resilient (> 2.5) based on their mean resilience. A majority (68.52%) of catchments, mainly in the central, central-eastern, western ghats, and southern regions, fall into the low (11.11%) and moderate (57.41%) resilience categories, while 31.48% of catchments demonstrate high resilience (Fig. 4). Subsequently, the resilience variability is assessed using the coefficient of variation, leading to a classification into low (< 0.75), moderate (0.75–1), and high (> 1) variability. Notably, 38.89% and 25.93% of catchments display moderate and high variability, respectively. Additionally, a strong inverse relationship is observed between mean resilience and resilience variability (ρ = − 0.90), suggesting that catchments with high mean resilience typically exhibit low variability and vice versa. Furthermore, trend analysis on the resilience index time series reveals that while 33.33% of catchments show a negative trend, the remaining 66.67% demonstrate a positive trend. And it is to be noted that 48.15% and 18.52% of catchments exhibit a moderate (0–0.10/year) and high trend magnitude (> 0.10/year), respectively.

## Discussion and conclusion

This study undertakes an evaluation of various hydroclimatic risks, specifically relating to floods, and resilience across 54 catchments in the peninsular region of India, during the period 1988 to 2011. To achieve this, initially, an assessment of extreme precipitation (R1 and R5) and discharge indices (Q1 and Q5) is conducted. Following this, return levels are estimated using Generalized Extreme Value (GEV) models. In order to account for non-stationarities in behaviour of extreme indices, the best model is chosen from among 26 non-stationary models and one stationary model, using the likelihood ratio test. It's important to note that global climate modes, namely ENSO, IOD, and AMO, are factored into the non-stationary models as covariates due to their potential influence on these extreme indices over the Indian subcontinent.

The results show that 66% of catchments, mainly in the central-eastern, southernmost western ghats, and parts of the western region, have moderate to high levels (> 900 mm and > 200 mm) of mean annual precipitation (prcptot) and mean annual discharge (Qtot), respectively. Furthermore, a positive trend is observed in prcptot/Qtot across 53.70%/40.74% of the catchments, with both indices sharing similar spatial trend patterns. For R1 and Q1, moderate to high mean values (> 70 mm and > 20 mm respectively) are evident in 70.37%/62.96% of the catchments, primarily in the central, central-eastern, southern western ghats, and some catchments in the western region. Positive trends for R1/Q1 are present in 51.85%/40.74% of the catchments, exhibiting high resemblance in their spatial trend patterns. Additionally, mean R5 and Q5 also show an identical spatial pattern where 74.07%/64.82% of catchments demonstrate moderate to high magnitudes (> 150 mm and > 50 mm), respectively, predominantly lying-in centre-eastern, southernmost western ghats and parts of western region. And R5/Q5 indicate positive trend in 53.70/40.74% catchments. In general, spatial pattern of trend statistics of R5 and Q5 exhibit high resemblance except in central-eastern region. Overall, the spatial patterns of mean R1, Q1, R5, Q5, and Qtot mirror those of the mean annual precipitation (mean prcptot) across the 54 catchments. This indicates that prcptot is the primary hydrological process influencing hydroclimatic extremes. Overall, the spatial patterns of mean R1, Q1, R5, Q5 and qtot show slight resemblance with mean annual average precipitation across the 54 catchments, which indicates that precipitation is main hydrological process driving hydroclimatic extremes as evident in recent literature^43–45^.

Previous research indicate that recent shifts in extreme precipitation and floods could be driven by natural variations^46,47^ and human-induced climate change^48,49^. A direct relationship has been established between precipitation and global climate modes in the Indian subcontinent in recent studies^50,51^. In this study, the best fit Generalized Extreme Value (GEV) models reveal that all extreme indices, namely R1, Q1, R5, and Q5, exhibit non-stationary behaviour. This indicates that extreme precipitation and flood events are not occurring at a constant rate or intensity over time but are instead varying in 54 catchments. This suggests that global climate modes substantially influence extreme precipitation and floods across 54 peninsular catchments. Further analysis of design return levels shows that 85.18% and 74.08% of catchments respectively exhibit moderate to high ^10yr^R1 and ^10yr^Q1 (> 100 mm/> 30 mm). Notably, the spatial patterns of mean R1, mean Q1, ^10yr^R1 and ^10yr^Q1 show strong resemblances to one another. Similarly, 70.37% of catchments exhibit moderate to high ^10yr^R5 and ^10yr^Q5 (> 220 mm and > 85 mm), and the spatial patterns of mean R5, mean Q5, ^10yr^R5, and ^10yr^Q5 also correspond closely with one another.

And, catchment resilience is quantified using convex model, whose input is annual discharge time-series. The annual discharge time series is obtained from simulated discharge output of best hydrological model across SVM, RVM, Random Forest and *abcd* model. The results of model performance for the hydrological models suggests that machine learning models perform better than conceptual hydrological model in simulating monthly discharge. Additionally, model performance of SVM and RVM is similar, however random forest outperforms other models. While machine learning models are capable for simulating single output variable, it lacks the simulation of sub-components of hydrological model such as groundwater storage, soil moisture and others. For such hydrological applications, coupling of physical processes along with machine learning, deep learning and artificial intelligence can be utilized to develop robust models^52–55^. Furthermore, the catchment resilience results show that around 68.52% catchments encompass low-moderate resilience, predominantly lying in central, central-eastern, western ghats and southern region.

While our study provides valuable insights into hydroclimatic extremes and resilience in peninsular Indian catchments, it also highlights areas for future research. First, we acknowledge that our large-scale focus may overlook microscale hydrological variations. Thus, future studies could aim for finer scale analyses to consider microclimate impacts and local heterogeneities^56^. Second, our application of linear combinations of global climate modes as covariates in GEV models is a limitation. Non-linear relationships or additional covariates could further illuminate the non-stationarity of hydroclimatic extremes^57,58^. Third, we employed a specific convex model for our resilience assessment. Using diverse resilience metrics or a composite resilience index may offer more nuanced insights. Moreover, we didn't incorporate local factors like temperature anomaly or relative humidity into our models, an aspect future studies might consider for improved model performance. While our risk assessment considers the hydroclimatic system's behaviour, socio-economic and ecological factors are overlooked. Therefore, a multi-disciplinary risk analysis that includes these aspects could offer a more holistic view of catchment vulnerability^59^.

The findings of this study can be directly implemented in socio-economic policy in several ways. These include bolstering infrastructure resilience, especially in catchments showing low to moderate resilience, to cope with increased hydroclimatic extremes. Emphasizing sustainable water management practices such as efficient irrigation systems could also help mitigate these risks. Furthermore, by understanding the influence of teleconnections (ENSO, IOD, and NAO) on extreme precipitation and discharge, policymakers can develop early warning systems to better predict and prepare for hydroclimatic extremes, thereby minimizing their negative impacts. Moreover, our study highlights the need for catchment-specific land use planning to proactively mitigate potential damages. Incorporating our findings into disaster management plans could provide more nuanced information, making these plans more effective.

## Methodology

### Study area and data

**Figure 5:**
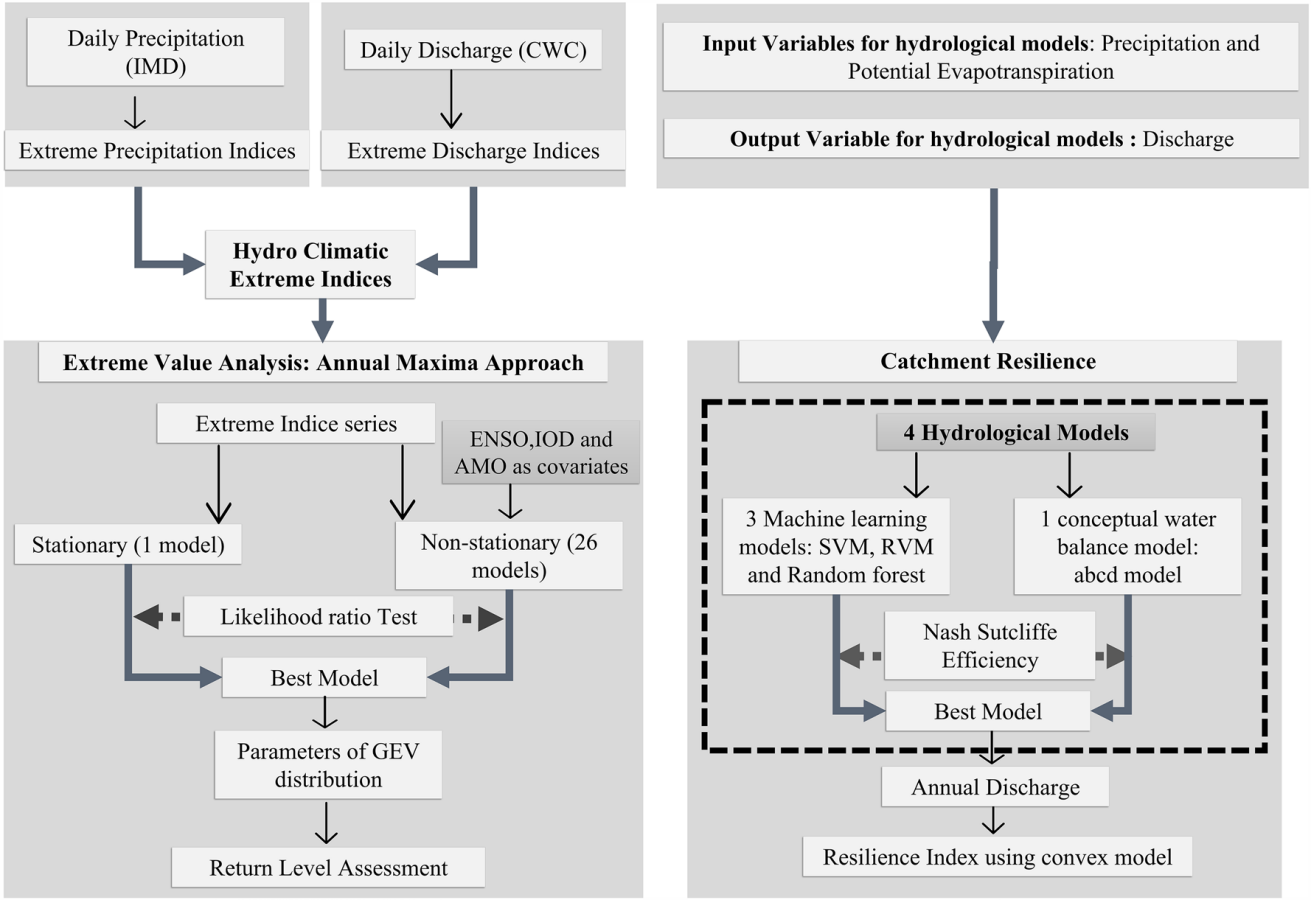
Schematic of method utilized for assessment of hydroclimatic extremes and resilience.

The present study (Fig. 5 shows outline of method used) is conducted for 54 catchments of Peninsular India, which lie in 14 different river basins of India (India-WRIS classification) and covers approximately 11.3% of country’s area^60^ as shown in Fig. S3. These sample catchments comprise of range of distinct catchment attributes such as catchment area (500–16,900 km^2^), annual precipitation (631–3931 mm) and annual discharge (Table. S1), representative of entire Peninsular region. This enabled for a comprehensive assessment of resilience and extremes in the region. Further, the hydroclimate variables used in study are precipitation, discharge and potential evapotranspiration. Daily precipitation data are obtained from India Meteorological Department (IMD)^20^ for the period from 1988 to 2011 at high spatial resolution (0.25° × 0.25°). This dataset incurs the ability to capture the spatial pattern of extreme and annual precipitation across India, and has been widely utilized in recent literature^35,61^. And, daily observed discharge data of 54 catchments are taken from India-WRIS (Water Resources Information System) portal (http://www.india-wris.nrsc.gov.in/), which provides discharge dataset from Central Water Commission (CWC), Govt. of India^60^. The data for potential evapotranspiration (PET) was sourced from the Climatic Research Unit (CRU) Time-series (TS) Version 4.01.This dataset provides monthly gridded PET at a high-resolution of 0.5 × 0.5°, covering the years 1901–2016^62^. The Penman–Monteith formula, which incorporates a variety of climatic factors such as average temperature, highest and lowest temperatures, vapour pressure, cloud cover, and wind speed, is used to calculate the PET for this dataset^62^.

### Hydroclimatic extreme indices

Extreme precipitation and discharge indices listed in Table 1, are calculated using daily precipitation and discharge timeseries for each catchment. The indices used here are developed by ETCCDMI-Expert Team on Climate Change Detection Monitoring and Indices^63^ and has been used widely in hydroclimate extreme assessment^64,65^. In this study, 3 precipitation extreme indices (R1, R5 and PRCPTOT) and their counterparts, 3 discharge extreme indices (Q1, Q5, and QTOT) are selected, firstly to understand the individual behaviour of precipitation and discharge extreme indices and most importantly to investigate the relationship between extreme precipitation indices and extreme discharge indices.

**Table 1:** Hydroclimate extremes indices.

Index	Definition	Unit	Indicator type
R1	Annual maximum 1-day precipitation	mm	Absolute
R5	Annual maximum consecutive 5-day precipitation	mm	Absolute
PRCPTOT	Annual total precipitation	mm	Absolute
Q1	Annual highest daily streamflow	mm	Absolute
Q5	Annual highest average discharge in consecutive 5 days	mm	Absolute
QTOT	Annual total discharge	mm	Absolute

### Global climate modes

For ENSO (El Niño-Southern Oscillation), Nino3.4 index is used, which is the averaged equatorial SST (Sea surface temperature) anomaly (5N-5S, 170W-120W) w.r.t 1981–2010^66^. Based on Nino3.4 index, El Niño or La Niña events are defined i.e., exceedance of greater than 0.4 °C or − 0.4 °C for consecutive six or more months. For IOD (Indian ocean dipole), dipole mode index (DMI) is used, which is defined as gradient in SST anomaly between western equatorial and southeastern equatorial Indian ocean. Generally, positive (negative) DMI represents warming (cooling) of western Indian ocean relative to eastern Indian ocean, which is connected to excess (deficit) rainfall over India^67,68^. For Atlantic Multidecadal oscillation (AMO), AMO index is used, which represents SST anomaly in North Atlantic Ocean^66^. Further, representative indice of global climate modes are obtained at monthly scale.

Floods in peninsular India are primarily driven by heavy summer monsoon rainfall, occurring from June to September (JJAS). Therefore, to align with these extreme conditions, global climate mode data is converted from monthly into seasonal (JJAS) time series. This data is then utilized as covariates in modelling extreme indices using GEV. Given that the block size used to separate extremes from daily precipitation/discharge series (for example, R1: the annual maximum 1 day precipitation) is one year, aggregation from monthly to seasonal format further streamlines the usage of these global climate modes as covariates in generalized extreme value models.

### Nonstationary framework

In this study, the Generalized Extreme Value (GEV) theory is used to investigate the influence of large-scale climate oscillations on four hydroclimatic extreme indices (R1, R5, Q1, and Q5) in peninsular India. These indices have been selected based on their adherence to the assumptions necessary for constructing a model using the GEV distribution^9^. To separate extreme events from the precipitation/discharge time series (daily or consecutive 5-day intervals), the block maxima method is employed, where each block represents one year. For instance, the R1 time series denotes the annual maximum 1-day precipitation derived from daily precipitation series, while the R5 series signifies the annual maximum precipitation over a consecutive 5-day period, derived from the consecutive 5-day precipitation series. The nonstationary analysis, predicated on the notion that fluctuations in precipitation/discharge extremes over a time period are influenced by individual or combined variations of global climate indices (ENSO, IOD, and AMO), assumes that hydroclimatic extreme data, represented by a continuous random variable, adheres to the GEV distribution.

Performance evaluation of several models is carried out: the original GEV model, the GEV models that includes a linearly varying location parameter, the GEV models with a linearly varying scale parameter, and the GEV models with both linearly varying location and scale parameters (Table. S2). The study uses an approach focused on linear relationships between global climate modes and hydroclimatic extremes due to their widespread use^69–71^ and easier interpretability in hydroclimatalogy.

1 $${X}_{t }\sim GEV\left(\mu \left(t\right),\sigma (t), \xi \right)$$

2 $$\mu \left(t\right)={ \beta }_{0}+ {\beta }_{1}\left(cov1\right)+ {\beta }_{2}\left(cov2\right)+\dots$$

3 $$\sigma \left(t\right)={ \gamma }_{0}+ {\gamma }_{1}\left(cov1\right)+ {\gamma }_{2}\left(cov2\right)+\dots$$

Equation 1 illustrates the GEV model, where location and scale parameters linearly vary as a function of covariates, which are global climate modes. Table S2 enumerates 26 distinct models (M1 to M26), constructed using different combinations of these covariates: ENSO, IOD, and AMO, denoted as C1, C2, and C3 respectively. To avoid modelling complexity, the shape parameter of the GEV distribution has been kept constant^9,72,73^. The cumulative probability distribution function of the G.E.V. distribution is given by Eq. (4)

4 $$F\left(x,\mu ,\sigma ,\xi \right)=\left\{\begin{array}{ll}\mathrm{exp}\{ -{\left[1+\xi \left(\frac{x-\mu }{\sigma }\right)\right]^{-1/\xi}} \}, & \quad \upsigma > 0, 1+ \xi \left(\frac{x-\mu } {\sigma }\right) > 0, \xi \ne 0 \\ \mathrm{exp}\left\{ -\mathrm{exp}\left[-\frac{\left(x-\mu \right)} {\sigma }\right]\right\}, & \quad \sigma > 0, \xi =0 \end{array}\right.$$

In Eq. (4), 'x' denotes the time series of extremes, while 'μ', 'σ', and 'ξ' symbolize the location, scale, and shape parameters of the GEV distribution, respectively. During stationary analysis, these parameters are held constant, while in nonstationary analysis, they are considered functions of covariates. These parameters can be determined through maximum likelihood estimation. Few examples of nonstationary GEV models presented in Table S2 are as follows:

5 $${\mathbf{M2}}: X\sim GEV\left[ {\upmu 0 + \upmu 2C2,\sigma ,\xi } \right]$$

6 $${\mathbf{M18}}: X\sim GEV\left[ {\left( {\upmu 0 + \upmu 1C1 + \upmu 2C2} \right),\left( {\sigma_{0} + \sigma 2C2 + \sigma_{3} C3} \right),\xi } \right]$$

In model M2, the GEV distribution's location parameter is a function of IOD (Eq. (5)). Conversely, model M18 incorporates ENSO, IOD, and AMO, allowing for variations in the GEV distribution's location and scale parameters (Eq. (6)). This implies that the distribution's scale parameter is influenced by IOD and AMO, while its location parameter is determined by ENSO and IOD. The remaining models are constructed in a similar manner. Table S2 enumerates all 27 models employed in the study, including one stationary (M0) and 26 nonstationary. Parameter estimates from the GEV distribution are derived via the maximum likelihood estimation (MLE) method, where the parameter set θ = [μ, σ, ξ] is determined at the maximum value of the likelihood function^74^.

Likelihood ratio (LR) tests have been conducted to evaluate their relevance of Nonstationarity in the data^9^. For nested models ${M}_{S}\subset {M}_{NS}$, the hypothesis testing of nonstationarity is carried out based on deviance statistic (*D*). The asymptotic distribution of D is supposed to have Chi-squared (${\chi }_{k}^{2}$) distribution with k degree of freedom^74 ^ (Eq. (7)).

7 $$\mathbf{D}=2[nllh(S) - nllh(NS)]$$

where nllh (S) and nllh (NS) are maximized log-likelihood under stationary and nonstationary models respectively. At a specific level of significance (5% in our case), derived values of D are compared to critical values from the ${\chi }_{k}^{2}$ distribution, where large values of D imply that the nonstationary model explains significantly better variation in the data than the stationary model. In case of nonstationarity, the best fit nonstationary model at a grid has been selected based on minimum p-value of LR test. MLE has been used to determine the parameters of the best fit GEV distribution (stationary or nonstationary). For detailed methodology, readers can refer to the flowchart presented in Fig. 5.

### Hydrological and machine learning models

In order to obtain catchment resilience, simulated annual discharge from aggregated monthly discharge is utilized from best performing models among *abcd* model, Random Forest, Support vector machine (SVM) and Relevance vector machine (RVM). The *abcd* model is a lumped conceptual hydrological model which takes inputs as precipitation, potential evapotranspiration and provides outputs such as soil moisture, groundwater storage, discharge^75^. It has 4 parameters *a,b,c* and* d*, which represent different catchment characteristics^75,76^. For more details on *abcd* model refer to Text S1. Random forest is a combination classification method founded on statistical learning theory by Breiman^77^. In this technique, resampling bootstrap method is utilized to draw multiple samples, and further classification trees are constructed for each sample. Finally, voting determines the final classification results through aggregation of all forecast classification trees. Furthermore, SVM is a new technique based on statistical learning theory and risk minimization hypothesis, and has proven to be efficient and robust algorithm for regression and classification^78–80^. And, RVM is also a powerful technique for classification and regression, based on the concept of sparse Bayesian learning^81,82^. Random forest, SVM and RVM have been used widely in field for hydrology for discharge forecasting, drought monitoring and other applications.

Here, input variable for these 4 models is precipitation and potential evapotranspiration and output variable is discharge at a monthly scale. The models are constructed for 54 peninsular catchments for the period 1988–2011, where calibration and validation period 1989–2004 and 2005–2011, respectively. And, Nash Sutcliffe Efficiency (NSE) is used as a measure of model performance which represents the relationship between observed and simulated discharge.^83^

### Catchment resilience

The catchment resilience is evaluated using a resilience index by the application of a convex model using the principle of critical slowing down. The method to calculate catchment resilience is adopted from^42^, where resilience index (${p}_{i}$) is based upon the temporal changes in the state variable. Here, we use annual discharge timeseries as the state variable to evaluate a time series of resilience index for understanding the temporal changes in catchment resilience over the study period. The system's resilience at a given point in time, denoted by i, is signified by the differences in ${x}_{i}$ and its “n” neighbouring points $({x}_{\left(i-n\right)},\dots .,{x}_{\left(i-2\right)},{x}_{\left(i-1\right)},{x}_{\left(i+1\right)},\dots .,{x}_{\left(i+n\right)}$. Greater differences denote higher resilience. Hence, we introduce an indicator ${p}_{i}$, which quantifies the number of adjacent points showing significant deviation from ${x}_{i}$. This metric, ${p}_{i}$, thus serves as a reflection of the system's resilience at the time i (Eq. 9). In the present study value n is taken as 4, and $\lambda$ = 0.75, as suggested in^42^.

8 $${g}_{i,j}=\left\{\begin{array}{cc}1& \left|{x}_{i}-{x}_{j}\right| \ge \varepsilon \\ 0& \left|{x}_{i}-{x}_{j}\right|< \varepsilon \end{array}\right\}, \;\;\varepsilon =0.5\lambda ({x}_{max}-{x}_{min})$$

9 $${p}_{i}=\sum_{j=i-n}^{j=i+n}{g}_{i,j}$$

where,$\varepsilon$ is threshold limit; ${x}_{max}$ and ${x}_{min}$ represent the maximum and minimum of state variable (x), ${p}_{i}$ resilience indicator for time i.

## Supplementary Information

Supplementary Information.

## Data Availability

Daily precipitation data, discharge and potential evapotranspiration (PET) data are obtained from India Meteorological Department (IMD) (Pai et al., 2014), India-WRIS (Water Resources Information System) portal (https://indiawris.gov.in/wris) and Climatic Research Unit (CRU) Time-series (TS) data version 4.01 data (CRU TS v. 4.01), respectively. Further, the data that support the findings of this study are available from the corresponding author on reasonable request.
