# Supplementary material for: Understanding non-stationarity of hydroclimatic extremes and resilience in Peninsular catchments, India
Source: Sci Rep. 2023 Aug 2;13:12524. doi: 10.1038/s41598-023-38771-w (PMC10397228; doi:10.1038/s41598-023-38771-w)
Supplement: Supplementary file 1 — Supplementary Information. [file 41598_2023_38771_MOESM1_ESM.docx]

**Understanding non-stationarity of hydroclimatic extremes and resilience in Peninsular catchments, India**

Nikhil Kumar^1^, Piyush Patel^2^, Shivam Singh^3^, Manish Kumar Goyal^4*^

^1,2,3 and 4^Department of Civil Engineering, Indian Institute of Technology, Indore-453552, India

(*Corresponding author: mkgoyal@iiti.ac.in)

**Fig. S1.** Percentage difference between stationary (ST) and best model (BM-only NS behaviour found) estimates$(\frac{ST-BM}{BM}\times100)$ for 10-year return level for hydroclimatic extremes (R1, Q1, R5 and Q5)

**Fig. S2.** Conceptual Framework of *abcd* hydrological model

**Fig. S3.** Study area, 54 catchments in peninsular India with (a) elevation from Shuttle Radar Topography Mission (Farr, 2007) and (b) mean annual precipitation

**Table. S1.** Annual mean of hydroclimatic variables (Precipitation ($\bar{\boldsymbol{P}}$), Potential Evapotranspiration ( $\bar{\boldsymbol{PE}}$) and Discharge ($\bar{\boldsymbol{Q}}$)) and area of catchments

**Table. S2.** List of total 27 models (1 stationary (M0) and 26 nonstationary) used in the study

**Text. S1.** “*abcd*” Model Hypothesis


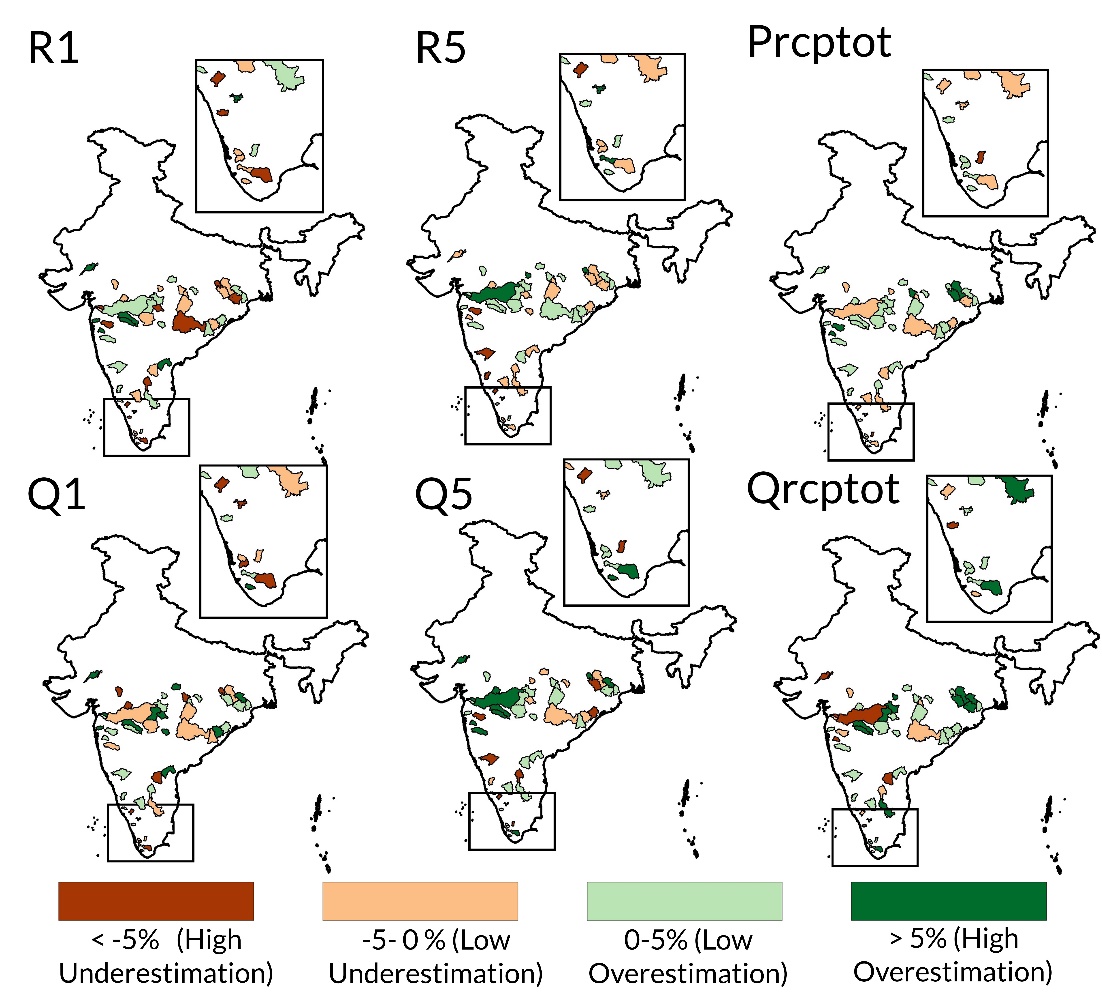


**Fig. S1.** Percentage difference between stationary (ST) and best model (BM-only NS behaviour found) estimates$(\frac{ST-BM}{BM}\times100)$ for 10-year return level for hydroclimatic extremes (R1, Q1, R5 and Q5). The maps were created using ArcGIS 10.7 software (https://www.esri.com/en-us/arcgis/products/arcgis-desktop/resources)

**Table. S1** Annual mean of hydroclimatic variables (Precipitation ($\bar{\boldsymbol{P}}$), Potential Evapotranspiration ( $\bar{\boldsymbol{PE}}$) and Discharge ($\bar{\boldsymbol{Q}}$)) and area of catchments

| Catchment  ID | River Basin | Catchment | Area (Km^2^) | Annual Mean (1989-2011) | | |
| --- | --- | --- | --- | --- | --- | --- |
|  |  |  |  | $\bar{\boldsymbol{P}}$ **(mm)** | $\bar{\boldsymbol{PE}}$ **(mm)** | $\bar{\boldsymbol{Q}}$ **(mm)** |
| 1 | Baitarani &  Brahmani | Anandapur | 8890.13 | 1542.88 | 1419.67 | 569.63 |
| 2 |  | Gomlai | 22340.54 | 1346.61 | 1415.86 | 497.04 |
| 3 |  | Jaraikela | 10708.86 | 1320.26 | 1395.69 | 419.78 |
| 4 |  | Tilga | 2684.78 | 1277.71 | 1341.77 | 738.66 |
| 5 | Cauvery | K.M.Vadi | 1454.40 | 1019.09 | 1457.30 | 238.01 |
| 6 |  | T.K.Halli | 8101.98 | 851.02 | 1559.78 | 92.34 |
| 7 |  | Thengumarahada | 673.26 | 1384.58 | 1422.54 | 499.05 |
| 8 | East Coast  Subzone | Kashinagar | 8074.27 | 1326.94 | 1458.57 | 355.86 |
| 9 |  | Purushottampur | 7154.39 | 1304.78 | 1484.54 | 305.15 |
| 10 |  | Srikakulam | 8633.54 | 1248.16 | 1483.79 | 302.22 |
| 11 | East Flowing  Rivers | Thammavaram | 7992.96 | 859.62 | 1787.60 | 128.70 |
| 12 |  | Vazhavachanur | 11594.07 | 784.73 | 1642.80 | 31.83 |
| 13 |  | A.P.Puram | 1286.37 | 826.77 | 1348.36 | 23.58 |
| 14 |  | Murappanadu | 2912.39 | 761.73 | 1443.95 | 194.93 |
| 15 |  | Theni | 899.24 | 865.79 | 1306.05 | 685.79 |
| 16 | Godavari | Bhatpalli | 3134.30 | 1136.42 | 1733.54 | 373.91 |
| 17 |  | Hivra | 10187.12 | 896.13 | 1738.50 | 152.47 |
| 18 |  | Nandgaon | 4527.22 | 1073.24 | 1745.90 | 200.52 |
| 19 |  | Pachegaon | 5554.25 | 730.58 | 1650.38 | 135.74 |
| 20 |  | Purna | 15119.35 | 804.66 | 1737.75 | 103.68 |
| 21 |  | Satrapur | 7398.06 | 1069.70 | 1672.20 | 270.55 |
| 22 |  | Zari | 5524.57 | 784.58 | 1750.37 | 96.39 |
| 23 |  | P.G.Bridge | 14093.19 | 975.88 | 1746.00 | 293.49 |
| 24 |  | Pathagudem | 39148.24 | 1469.94 | 1562.00 | 597.71 |
| 25 | Krishna | Cholachguda | 9897.49 | 690.14 | 1654.65 | 94.81 |
| 26 |  | Phulgaon | 2117.59 | 1537.28 | 1531.78 | 621.39 |
| 27 |  | Sarati | 6754.84 | 882.95 | 1558.10 | 819.55 |
| 28 |  | Shimoga | 2727.49 | 2306.32 | 1499.78 | 2029.66 |
| 29 | Mahanadi | Andhiyarkore | 2141.48 | 1052.88 | 1538.61 | 155.15 |
| 30 |  | Ghatora | 2980.72 | 1240.73 | 1554.33 | 300.72 |
| 31 |  | Kotni | 7012.96 | 1215.94 | 1626.34 | 285.04 |
| 32 |  | Pathardhi | 2508.82 | 1174.85 | 1635.01 | 407.18 |
| 33 |  | Simga | 16906.83 | 1164.50 | 1630.80 | 288.56 |
| 34 | Mahi | Mataji | 3968.57 | 960.35 | 1824.28 | 345.75 |
| 35 | Narmada | Gadarwara | 2212.96 | 1126.07 | 1555.05 | 645.03 |
| 36 |  | Kogaon | 3887.82 | 792.41 | 1807.18 | 303.42 |
| 37 |  | Mohgaon | 4017.72 | 1209.50 | 1507.03 | 598.18 |
| 38 |  | Patan | 4038.50 | 1247.06 | 1490.88 | 425.30 |
| 39 | Pennar | Alladupalli | 8697.70 | 806.76 | 1837.54 | 194.27 |
| 40 |  | Singavaram | 5921.54 | 631.22 | 1721.09 | 31.49 |
| 41 | Sabarmati | Kheroj | 878.14 | 732.74 | 1776.41 | 431.23 |
| 42 | Subanarekha | Adityapur | 6416.67 | 1410.01 | 1389.64 | 454.35 |
| 43 |  | Govindpur | 4324.76 | 1660.38 | 1420.99 | 755.49 |
| 44 | Tapi Basin | Gidhade | 52134.18 | 813.61 | 1725.01 | 131.05 |
| 45 | West Flowing  Rivers | Kamalpur | 5011.10 | 741.61 | 1841.81 | 73.78 |
| 46 |  | Ayilam | 502.04 | 1583.61 | 1387.67 | 1392.52 |
| 47 |  | Durvesh | 1990.40 | 2775.45 | 1620.63 | 1639.27 |
| 48 |  | Erinjipuzha | 852.84 | 3931.04 | 1466.11 | 2715.24 |
| 49 |  | Kallooppara | 697.30 | 3730.53 | 1418.63 | 2551.65 |
| 50 |  | Karathodu | 770.12 | 2394.60 | 1507.95 | 1715.15 |
| 51 |  | Kidangoor | 592.58 | 3311.19 | 1411.73 | 2919.04 |
| 52 |  | Mahuwa | 1712.58 | 1413.37 | 1703.51 | 823.77 |
| 53 |  | Thumpamon | 814.04 | 2912.71 | 1425.53 | 1460.45 |
| 54 |  | Gadat | 1465.97 | 1833.66 | 1651.63 | 1112.58 |

**Table S2.** List of total 27 models (1 stationary -M0 and 26 nonstationary GEV models) used in the study

| **Model ID** | **Description** | **Covariates: Associated Global Climate Modes** |
| --- | --- | --- |
| M0 | *X ̴̴̴ GEV[ µ, σ, ξ]* |  |
| M1 | *X ̴̴̴ GEV[ µ_0_ +µ1c1, σ, ξ]* | ENSO |
| M2 | *X ̴̴̴ GEV[ µ_0_ +µ2c2, σ, ξ]* | IOD |
| M3 | *X ̴̴̴ GEV[ µ_0_ +µ3c3, σ, ξ]* | AMO |
| M4 | *X ̴̴̴ GEV[ µ_0_ +µ1c1 _+_ µ2c2 , σ, ξ]* | ENSO+ IOD |
| M5 | *X ̴̴̴ GEV[ µ_0_ +µ2c2 _+_ µ3c3 , σ, ξ]* | IOD+ AMO |
| M6 | *X ̴̴̴ GEV[ µ_0_ +µ3c3 _+_ µ1c1 , σ, ξ]* | AMO+ ENSO |
| M7 | *X ̴̴̴ GEV[ µ_0_ +µ1c1 _+_ µ2c2 + µ3c3 , σ, ξ]* | ENSO+ IOD+ AMO |
| M8 | *X ̴̴̴ GEV[( µ_0_ +µ1c1_)_, (σ_0_ + σ_1_C_1_), ξ]* | ENSO, ENSO |
| M9 | *X ̴̴̴ GEV[( µ_0_ +µ1c1_)_, (σ_0_ + σ2C2), ξ]* | ENSO, IOD |
| M10 | *X ̴̴̴ GEV[( µ_0_ +µ1c1_)_, (σ_0_ + σ3C3), ξ]* | ENSO, AMO |
| M11 | *X ̴̴̴ GEV[( µ_0_ +µ2c2_)_, (σ_0_ + σ1C1), ξ]* | IOD, ENSO |
| M12 | *X ̴̴̴ GEV[( µ_0_ +µ2c2_)_, (σ_0_ + σ_2_C_2_), ξ]* | IOD, IOD |
| M13 | *X ̴̴̴ GEV[( µ_0_ +µ2c2_)_, (σ_0_ + σ3C3), ξ]* | IOD, AMO |
| M14 | *X ̴̴̴ GEV[( µ_0_ +µ3c3_)_, (σ_0_ + σ1C1), ξ]* | AMO, ENSO |
| M15 | *X ̴̴̴ GEV[( µ_0_ +µ3c3_)_, (σ_0_ + σ2C2), ξ]* | AMO, IOD |
| M16 | *X ̴̴̴ GEV[( µ_0_ +µ3c3_)_, (σ_0_ + σ_3_C_3_), ξ]* | AMO, AMO |
| M17 | *X ̴̴̴ GEV[( µ_0_ +µ1c1 _+_ µ2c2_)_, (σ_0_ + σ_1_C_1_+ σ_2_C_2_), ξ]* | ENSO+ IOD, ENSO+ IOD |
| M18 | *X ̴̴̴ GEV[( µ_0_ +µ1c1 _+_ µ2c2_)_, (σ_0_ + σ2C2+ σ3C3), ξ]* | ENSO+ IOD, IOD+ AMO |
| M19 | *X ̴̴̴ GEV[( µ_0_ +µ1c1 _+_ µ2c2_)_, (σ_0_ + σ3C3+ σ1C1), ξ]* | ENSO+ IOD, AMO+ ENSO |
| M20 | *X ̴̴̴ GEV[( µ_0_ +µ2c2 _+_ µ3c3_)_, (σ_0_ + σ_2_C_2_+ σ1C1), ξ]* | IOD+ AMO, IOD+ ENSO |
| M21 | *X ̴̴̴ GEV[( µ_0_ +µ2c2 _+_ µ3c3_)_, (σ_0_ + σ_2_C_2_+ σ_3_C_3_), ξ]* | IOD+ AMO, IOD+ AMO |
| M22 | *X ̴̴̴ GEV[( µ_0_ +µ2c2 _+_ µ3c3_)_, (σ_0_ + σ3C3+ σ1C1), ξ]* | IOD+ AMO, AMO+ ENSO |
| M23 | *X ̴̴̴ GEV[( µ_0_ +µ3c3 _+_ µ1c1_)_, (σ_0_ + σ1C1+ σ2C2), ξ]* | AMO+ ENSO, ENSO+ IOD |
| M24 | *X ̴̴̴ GEV[( µ_0_ +µ3c3 _+_ µ3c1_)_, (σ_0_ + σ2C2+ σ3C3), ξ]* | AMO+ ENSO, IOD+ AMO |
| M25 | *X ̴̴̴ GEV[( µ_0_ +µ3c3 _+_ µ1c1_)_, (σ_0_ + σ3C3+ σ1C1), ξ]* | AMO+ ENSO, AMO+ ENSO |
| M26 | *X ̴̴̴ GEV[( µ_0_ +µ1c1 _+_ µ2c2 + µ3c3_)_, (σ0 + σ1C1+ σ2C2+ σ3C3), ξ]* | ENSO+ IOD+ AMO, ENSO+ IOD+ AMO |

C1- ENSO (SST Nino 3.4)

C2 – IOD

C3 – AMO

**Text. S1**.  “*abcd*” Model Hypothesis

The *abcd* model, based on the Thornthwaite’s water balance structure^1^, uses precipitation and potential evapotranspiration to generate soil moisture, groundwater storage, and discharge data (Fig. S2). It employs four adjustable parameters reflecting catchment characteristics, which can vary due to land use changes or new water installations. While more complex models may improve predictions, they need extensive field data and significant calibration.

The *abcd* model utilizes four distinct parameters: *a, b, c,* and *d*, each corresponding to a unique catchment characteristic. Parameter '*a*' (0 ≤ *a* ≤ 1) signifies predisposition for discharge before soil saturation, '*b*' sets the upper limit for soil moisture storage plus evapotranspiration, '*c*' (0 ≤ *c* ≤ 1) represents the groundwater recharge to direct runoff ratio, while the inverse of '*d*' (0 ≤ *d* ≤ 1) indicates water's residence time in groundwater storage^2,3^ .The model uses the continuity equation for soil moisture storage, leading to

${(P}_{t}+S_{t-1})={(E}_{t}+S_{t})+GR_{t}+DR_{t} (S1)$

where$P_{t}$,$E_{t}$,$DR_{t}$ and $GR_{t}$are precipitation, actual evapotranspiration, direct runoff and groundwater recharge for the month respectively, and $S_{t}$and$S_{t-1}$ are soil moisture storage at the end of the present and preceding month, respectively. The elements of eqn (S1) are identified as the model's two state variables, available water (${W_{t}=P}_{t}+S_{t-1})$ and evapotranspiration opportunity ($Y_{t}=E_{t}+S_{t}$)^2^, under the presumption of a non-linear connection between them, expressed as follows:

$Y\left( W_{t} \right)=\frac{W_{t}+b}{2a}-\sqrt{\left( \frac{W_{t}+b}{2a} \right)^{2}-\frac{W_{t}b}{a}}$ (S2)


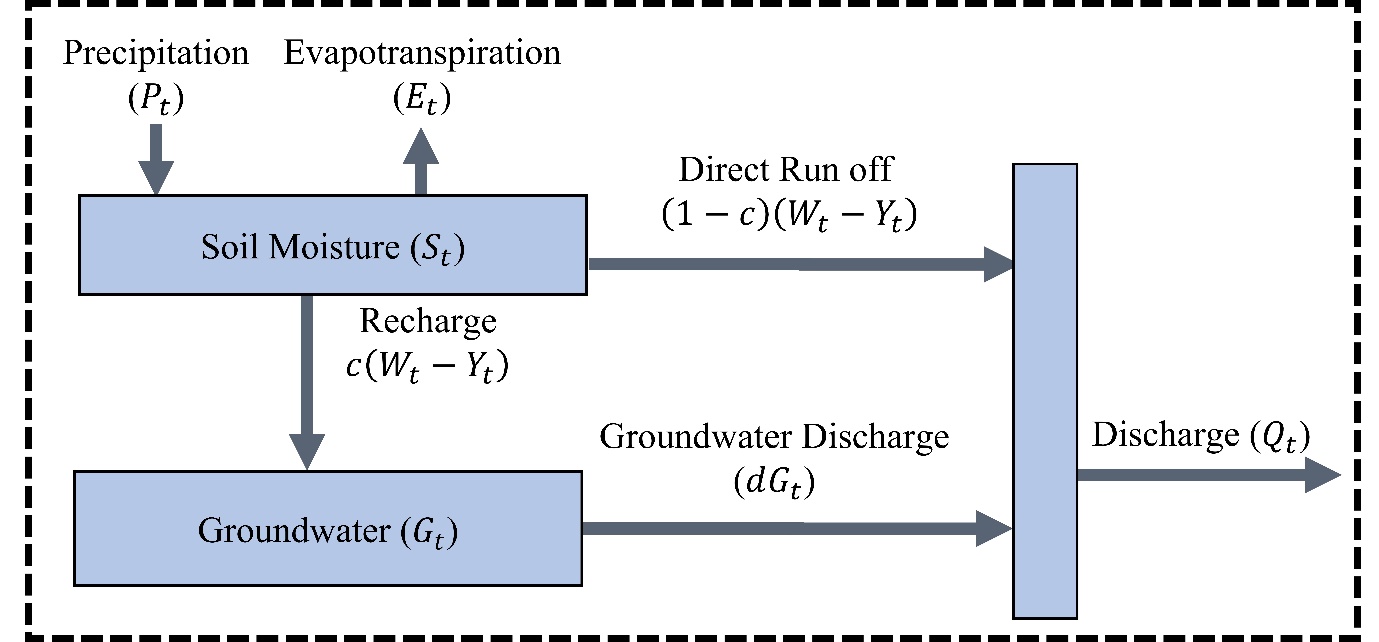


**Fig. S2.** Conceptual Framework of *abcd* hydrological model

Evapotranspiration opportunity ($Y_{t}$) refers to the highest potential evapotranspiration in a catchment for a specific period. The model assumes a direct proportionality between rate of soil moisture loss and potential evapotranspiration^3^, resulting in the expression, $S_{t}=Y_{t}exp\left( \frac{-PE_{t}}{b} \right)$. This expression and ($Y_{t}$) further simplify the calculation of actual evapotranspiration ($E_{t}$), as defined by eqn (S3).

$E_{t}=Y_{t}\left( 1-exp\left( \frac{-PE_{t}}{b} \right) \right)$ (S3)

Parameter *c* apportions $\left( W_{t}-Y_{t} \right)$ into groundwater recharge ($GR_{t}=c\left( W_{t}-Y_{t} \right)$) and direct runoff ($DR_{t}=\left( 1-c \right)\left( W_{t}-Y_{t} \right)$), while parameter d distributes the groundwater storage to groundwater discharge ($GD_{t}=dG_{t}$). Applying the continuity equation to groundwater storage results in $G_{t}=\frac{1}{1+d}\left( G_{t-1}+GR_{t} \right).$ The model's discharge is then calculated as the aggregate of groundwater discharge and direct runoff (eqn S4).

$Q_{t}=\left( 1-c \right)\left( W_{t}-Y_{t} \right)+dG_{t}$ (S4)


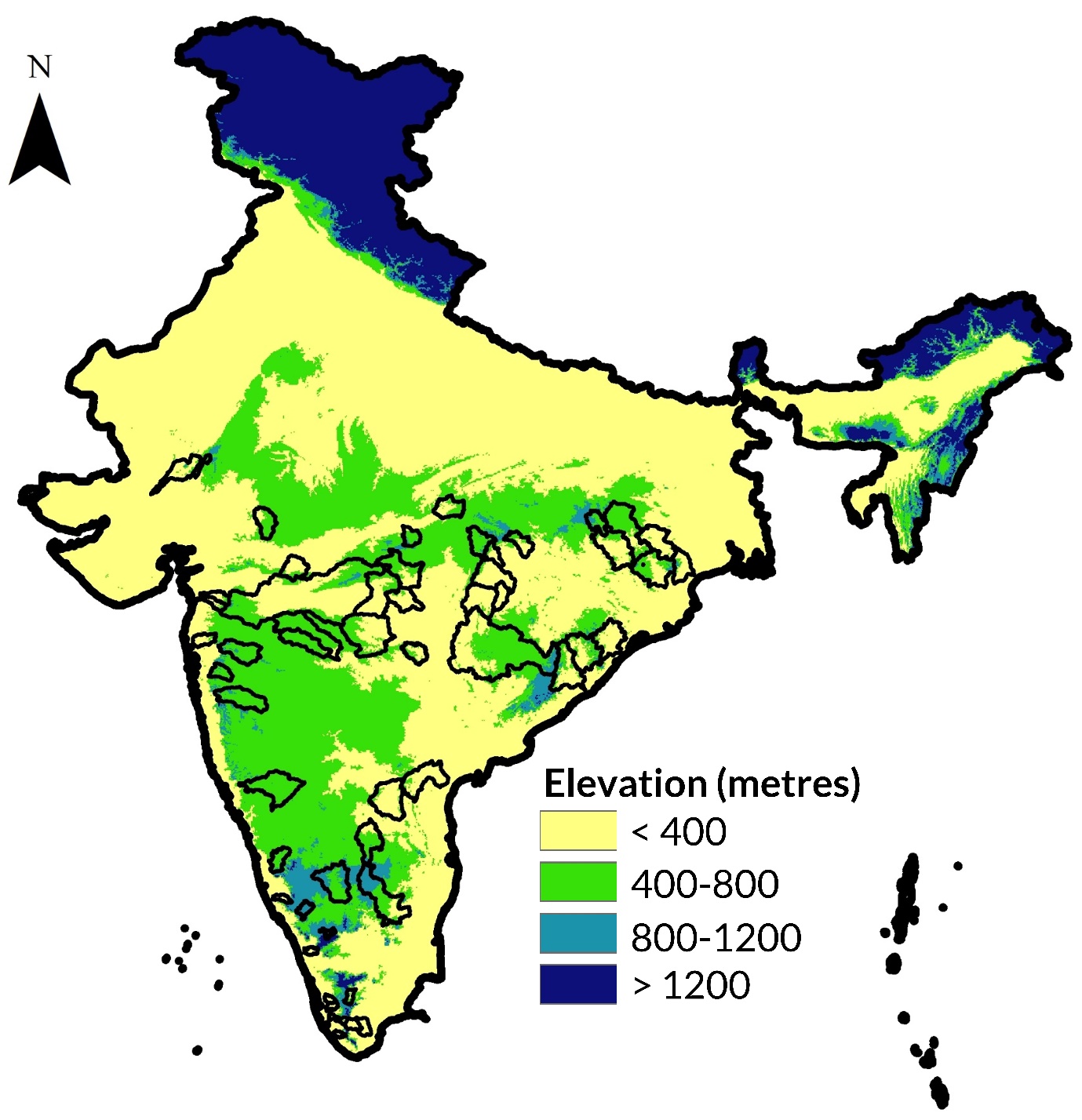


**Fig. S3.** Study area, 54 catchments in peninsular India with Elevation from Shuttle Radar Topography Mission^4^. The map was created using ArcGIS 10.7 software (https://www.esri.com/en-us/arcgis/products/arcgis-desktop/resources)

**References**

1. Thornthwaite, C. W. An approach toward a rational classification of climate. *Geogr. Rev.* **38**, 55–94 (1948).

2. Sankarasubramanian, A. & Vogel, R. M. Annual hydroclimatology of the United States. *Water Resour. Res.* **38**, 11–19 (2002).

3. Thomas Jr, H. A. Improved Methods for National tvater Assessment Water Resources Contract: WR15249270. *US Water Resour. Counc. Washington, DC, USA* (1981).

4. Farr, T. G. The shuttle radar topography mission: Reviews of Geophys., 45. *RG2004* 1–13 (2007).
